# Proceedings of the International Hahnemannian Association

**Published:** 1889-07

**Authors:** S. A. Kimball, R. Hearn

**Affiliations:** Secretary; Assistant Secretary


### HOMŒOPATHIC PHYSICIAN,

A MONTHLY JOURNAL OF

HOMŒOPATHIC MATERIA MEDICA AND CLINICAL MEDICINE.

If our school ever give up the strict inductive method of Hahnemann, we are lost, and deserve only to be mentioned as a caricature in the history of medicine."—CONSTANTINE HERING.

Vol. IX. JULY, 1889. No. 7.

### PROCEEDINGS OF THE INTERNATIONAL HAHNEMANNIAN ASSOCIATION.

JUNE 18TH-20TH, 1889.

The tenth annual meeting of the International Hahnemannian Association was opened by the President, Dr. Wm. A. Hawley, June 18th, 1889. This meeting was the first one held out of the United States; Toronto was selected for this meeting as a tribute to the Canadian and other foreign members. This Association is international in its membership and catholic in its purpose—the support of truth in medicine.

It may be well for us to briefly recall, at this time, the history of this Association, for many, unacquainted with its earlier history and the causes which led to its organization, may wonder why there are *two* general associations of homœopathic physicians in this country. The reply to this supposed query might be tersely stated thus: The International Hahnemannian Association was started to carry on the work which the American Institute had neglected, namely, the study of Hahnemann's *Organon* and of the *homœopathic* materia medica. The Institute was also organized for this very same work; but, as it grew in membership, it also became more and more eclectic in its work, until, finally, little or no homœopathic work was done at its meetings. Any one who doubts this assertion may easily verify it by looking over the volumes of the Institute's annual proceedings. The first meeting of the I. H. A. was held June 16th, 1880, at Milwaukee; the venerable Dr. P. P. Wells was chairman; Drs. Pearson, Berridge, Foote, and Pomeroy were appointed a committee upon organization. The next day the Association was organized by the following physicians: Drs. Ad. Lippe, G. F. Foote, C. Pearson (all three now dead), H. C. Allen, O. P. Baer, P. P. Wells, E. W. Berridge, W. H. Leonard, T. F. Pomeroy, J. P. Mills, E. Rushmore, T. F. Smith, E. A. Ballard, T. P. Wilson, T. W. Poulson, and E. Cranch. That the I. H. A. *has* been the centre of a strong influence for creating an interest in the study of the *Organon* and of the homœopathic materia medica is very evident. One can see this in the character of the work done by our medical societies in the last few years, as contrasted with their previous work; the same change is noted in many of the journals; it is shown in the organization of numerous clubs for the study of the *Organon* and of the materia medica. It may be safely asserted that the organization of the I. H. A. was the beginning of a reformation in American Homœopathy, and that the Association is not only doing a good work itself, but is influencing other societies in the right direction.

The brief report which we give of the tenth meeting shows that the members of the I. H. A. are still working for the philosophy and practice of medicine as taught by Samuel Hahnemann.

The first session was held in the Educational Department of the Normal School, and opened at 2.43 P. M., the President, *Dr. Wm. A. Hawley, in the chair.*

The following gentlemen were present at the opening session: Drs. Wm. A. Hawley, J. T. Kent, W. J. H. Emory, E. T. Adams, S. A. Kimball, F. W. Payne, H. Hitchcock, E. A. Ballard, H. C. Allen, J. V. Allen, E. W. Sawyer, Wm. P. Wesselhœft, C. W. Butler, S. Seward, J. B. Bell, B. L. B. Baylies, T. M. Dillingham, S. Long, Julius Schmitt, Allan B. Carr, J. A. Biegler, W. A. Foster, Mary F. Taft, H. H. Cobb, Dutton, McDonald, Sargent, Wm. L. Reed, L. H. Evans, J. D. Tyrrell, T. D. Stow, E. B. Nash, T. S. Hoyne, J. B. G. Custis, M. Preston, Wm. J. Guernsey, A. B. Eadie.

Dr. Wm. A. Hawley opened the session by reading the Presidential address, in which he recommended *incorporation* of the I. H. A. Dr. J. T. Kent in discussion opposed it, while Drs. Bell, Dillingham, and Butler with the President advised that it be carried into effect. The address was referred to the Publishing Committee after some further remarks.

Dr. Emory suggested that the Toronto papers be furnished with daily condensed reports of the proceedings. Dr. Kent moved that Drs. Emory, E. T. Adams, and A. B. Eadie be appointed as a committee. *Carried.*

*Report of Treasurer, Dr. J. D. Tyrrell,* was postponed, Dr. Tyrrell not being in the room.

In his report, Dr. Kimball, the Secretary, mentioned the reception of letters *from Paris Exposition,* Homœopathic Congress inviting delegates.

*Resignation* of Dr. J. F. Miller.

Dr. Butler moved that Dr. Miller's resignation be accepted. *Carried.*

Report of Treasurer read; debt five hundred and forty-seven dollars reduced to three hundred and seventy-two dollars.

Dr. H. C. Allen moved the report be referred to the *Auditing Committee,* Drs. Hitchcock, Schmitt, and Carr. *Carried.*

*Unfinished Business.*—Secretary refers to Dr. Hussey's resolution to change the by-laws of last year, so that the Bureau of Obstetrics and Diseases of Women and Children should be divided and the *Diseases of Women and Children* be transferred to the Bureau of *Clinical Medicine,* and the Bureau of Obstetrics contain that subject alone.

Dr. Butler asked if it could be made a special bureau?

Dr. Kimball replied in favor of that.

Dr. H. C. Allen opposed the resolution, and said we would be in as bad a position as the A. I. H.

Dr. Butler suggested that in that case we would also have a Bureau of Pedology, and all kinds of things.

Motion that the resolution be laid on the table. *Carried.*

Secretary received a resolution from Dr. Clark that the meeting of the Association be held in August. Referred over to next year.

Dr. Allen moved that the session be held to-night at Queen's Hotel, to-morrow morning at Normal School, after at Queen's. *Carried.*

Dr. Ballard moved a vote of thanks to the authorities for the use of this hall. *Carried.*

Dr. Ballard objected to the resolution passed last year, of prohibiting members from serving on more than one bureau (discussion). Resolution rescinded.

*Report of Board of Censors.*—Dr. Biegler, Chairman, reports the following gentlemen recommended by the Board of Censors to the membership of this Society. (The Secretary casting vote for each): B. M. Banerjee, M. D., Calcutta, India; S. W. Cohen, M. D., Waco, Texas; Isaiah Dever, M. D., Clinton, N. Y.;

A. B. Eadie, M. D., Toronto; Robert Farley, M. D., Phœnixville, Pa.; W. H. A. Fitz, Philadelphia, Pa.; Rolla C. Grant, M. D., Rochester, N. Y.; R. E. Jamieson, M. D., Jamaica Plains, Mass.; Mary F. Taft, M. D., Middletown, Conn.; J. W. Thatcher, M. D., Philadelphia, Pa.; J. A. Tomhagen, M. D., Sloan's Valley, Ky.

Dr. Biegler remarked that a number of applicants had not complied with the rules of the Association in sending a "thesis" (this closed the bureau).

*Bureau of Homœopathics.*—(Chairman, Dr. Wm. P. Wesselhœft.) Dr. Wesselhœft being absent, President requested Dr. Kent to act in his stead. First paper read was one by Dr. Wm. P. Wesselhœft, entitled "Practical Hints in the Management of Chronic Cases" (read by Dr. Wesselhoeft, Jr.).

Discussion by Drs. Butler, Bell, Long, H. C. Allen, Emory, Kent, Reed, J. V. Allen, Hitchcock.

Dr. Biegler advised one not to repeat the remedy as long as the patient's *mental* symptoms are improving, even though there may be aggravation of *physical* ones.

Drs. Butler and Reed were not agreed as to that.

Dr. Emory referred to homœopathic treatment of rheumatism; never knew organic heart disease follow it. Drs. Sawyer, Schmitt, and Ballard also took part in discussion, at the close of which Dr. Butler moved the adjournment of the session, to meet again at eight P. M. at the Queen's Hotel. *Carried.*

Meeting called to order at eight P. M., Dr. Hawley (President) in the chair.

President announced the Chairmen of Bureaus for ensuing year as follows:

Bureau of "Homœopathics"—C. W. Butler, M. D.

" "Materia Medica Provings"—W. L. Reed, M. D.

" "Clinical Medicine"—Julius Schmitt, M. D.

" "Surgery"—Thos. M. Dillingham, M. D.

" "Obstetrics"—W. J. H. Emory, M. D.

Secretary read telegram from Dr. Gee, of Chicago, regretting his absence, and letter from Dr. W. H. Leonard also regretting absence, and inviting Association to Minneapolis.

Bureau of *Homœopathics* reopened.

Dr. Kent presented second paper of Bureau by Dr. Wells, "The Revolution of Old School Physic." In the absence of Dr. Wells, paper was read by Mrs. Leberry. The paper confutes the Germ Theory and elicted discussion by Dr. Bell.

Paper by Dr. Hitchcock, "First Section of the *Organon*."

Paper by Dr. McNeil, of San Francisco, "*Genus Epidemicus*." Referred to Committee on Publication (read by title).

Paper by Dr. Nash, "Interrogations in Homœopathics." Dr. Nash being absent, paper referred to Committee on Publication, but afterward read by Dr. Nash next day.

Paper by Dr. Kent, "The Healing Principle." (The paper deals principally with idiosyncrasies); also gives theory of Rhus-tox. high curing rhus poisoning, etc.

Discussion by Dr. Long (says diseases are not contagious), Dr. Biegler (the contagion is the disease), Drs. Sawyer, Reed, Butler, H. C. Allen, Emory, Kent, Baylies, Kimball, J. V. Allen, and Ballard. (The discussion was very lengthy, and brought out many points of interest, such as treatment of toxical cases with high potencies of the same remedy, etc.) This paper closed the Bureau of "Homœopathics."

Motion to adjourn until ten A. M. next morning. *Carried.*

June 19th, ten A. M.—Session opened in the Educational Department of Normal School (Dr. Hawley in chair).

Address of welcome by Minister of Education, G. W. Ross; replied to by President.

Bureau of Homœopathics was re-opened to hear Dr. Nash's paper read, "Interrogations in Homœopathics."

Discussion by Drs. Butler, H. C. Allen, Stow, Nash, Kimball, Kent.

Dr. Biegler thought Dr. Nash's questions were best answered by turning to the *Organon.*

The discussion was a very exhaustive one, and the concensus of opinion pointed to the *Organon* for the answers to the paper.

Bureau of "Homœopathics" was then closed.

Committee on *President's address* reported (Dr. Kent in chair): Motion that the Association be incorporated, and committee appointed for that purpose. *Carried.*

Dr. Ballard suggested that, hereafter, the President's address be read as well to the laity; Dr. Kent objected, since the meeting was open to the public.

Treasurer's report read by Dr. Tyrrell, showing indebtedness of three hundred and forty-two dollars and seventy-two cents, and there was much discussion as to how to wipe out the debt. Report referred to the Auditing Committee.

Motion by Dr. Long, that when any member of this organization does not pay his dues within six months, the Treasurer draw on him at sight for the amount, and, if he refuses it, he be dropped from the membership of this Society. Motion was lost on division.

Motion for adjournment to meet at two P. M. in Queen's.

June 19th, two P. M.—President, Dr. Hawley, in chair.

Secretary announced proposition from Dr. H. C. Allen to print the "Transactions" in the *Advance*, as a supplement, so as to be bound separately afterward, for the cost of printing.

Moved and seconded that this offer be accepted. *Carried.*

*Bureau of Surgery*—Dr. Bell, Chairman.

Dr. Bell opened the Bureau by reading his paper, entitled "Histerism," which was very exhaustive and dealt well with the subject.

The paper was recommended by the Association to be printed in pamphlet form for widespread circulation (Dr. Allen offering to do the work).

Discussion by Drs. Stow, Custis, Ballard, Schmitt, and Long, all bearing testimony to the wonderful effects attained in the healing of wounds by homœopathic treatment instead of histerism. Antiseptics were entirely condemned by all.

Paper by Dr. Dillingham upon "Facts in Surgery."

Discussion by Drs. Dillingham: Don't use Calendula in wounds, unless indicated, not as Carbolic acid is used; use only hot water, the results are just as good. Dr. Hoyne always uses cold water, even in suppurations. Dr. Bell believed Dr. Dillingham was right, and promised to present at some future time report of thirty-six cases, three months' work in "Aseptic Surgery." In these cases the points observed were absolute cleanliness, perfect coaptation of parts, and rest of wounded part.

Paper by Dr. Stow, "Periorchitis with Abscess," and "a case involving amputation."

First case read by title and referred. The second case was read by Dr. Stow and discussed by Drs. Bell, Stow, and H. C. Allen.

Paper by Dr. McNeil, "Surgical Cases," was read by title and referred to Publishing Committee.

Paper by Dr. Thompson, New York, "Epulis," read by title and referred to committee.

Paper by Dr. Geo. H. Clark, "Sodium Ethylate in the treatment of Nævi," read and referred.

Paper by Dr. Campbell, "In praise of Calendula."

Discussion by Drs. Bell, H. C. Allen, Dillingham (Arnica, Calendula, and Hypericum for wounds, but used according to indications and not together as Dr. Campbell did in her case; Calendula for clean-cut wounds, Arnica for bruised and torn wounds); also Drs. Kimball, Nash, Schmitt, Long, Custis, Cobb, Stow, Biegler (had used these remedies in highly diluted form as local applications to wounds with good results).

Motion to adjourn, by Dr. Reed, until eight P. M. *Carried.*

Evening Session—eight P. M. (June 19th), President in chair. The first part of the proceedings was the reading by the Secretary of a report from the "Women's Homœopathic Association of Pennsylvania."

Motion that this report be accepted. *Carried.*

Dr. Bell—I think we ought to give some form of recognition to our friends in Rochester for their action in establishing a Hahnemannian Hospital.

Motion by Dr. Bell, seconded by Dr. Kent, that a committee of three be appointed to draft a resolution expressing the sentiments of this Association in regard to the action of our brethren in Rochester in establishing a Hahnemannian Hospital in that city. Drs. Bell, Kent, and Allen were appointed upon the committee.

The President appointed a committee, consisting of Drs. Wesselhœft, Bell, and Kimball to attend to the incorporation of this Association.

Bureau of Materia Medica—Dr. Ballard, Chairman of Bureau, presented the following papers: "Sanicula," by Dr. Gundlach; "Cantharides and Comparative Remedies," by Dr. J. V. Allen; "Verifications of Sanicula," by Dr. W. J. Guernsey; "Proving of Cocoaine," by Flora A. Waddell, M. D. (This paper was an excellent proving of the drug.)

J. V. Allen read his paper on "The Urinary Symptoms of Cantharides," in which he compared it with many other remedies of same class, as Cann-sat., Lycopod., Hydrangea, Apis, Copaiba, Tarent., etc., giving the special indications for each remedy, with their points of difference. A good paper.

Discussed by Drs. Bell, H. C. Allen, Butler, and Kent, who all praised the paper very highly.

Dr. Ballard—I have a collection of all the symptoms of Lac caninum, by Dr. Berridge; he gives 1,009.

Paper by Dr. H. C. Allen—"Dr. Wesselhœft's proving of *Mag-phos.*" Also that by Dr. Taft, of the *CM* potency by olfaction, she being very susceptible to the action of that drug. Also a proving by one of Dr. Campbell's patients and Dr. Holmes, Sycamore, Ill.

Discussion by Drs. Bell, Kent (gave case of aggravation from Mag-phos. in a lady, producing among other symptoms a terrible cough which nothing could stop until he antidoted the drug by a dose of Lachesis), also Drs. Nash, Biegler, Campbell, Reed, Ballard, and J. V. Allen.

Dr. H. C. Allen also read a paper on the proving of Mellilotus, giving some mental symptoms, principally insomnia. Dr. Nash had considerable experience in its use.

Dr. Kent moved for the election of officers first thing in the morning. *Carried.*

Dr. Butler arose to a question of privilege, and, in a very suitably-worded address, presented Dr. E. A. Ballard, Chairman of Bureau of Materia Medica, with a gold-headed cane, on behalf of the Association.

Motion by Dr. Allen, to adjourn till ten A. M. next morning. *Carried.*

Thursday, June 20th, 10.15 A. M.—Session re-opened. Dr. Hawley in the chair.

Election of officers for ensuing year.

Motion to proceed by Dr. Kent.

Dr. Butler nominated Dr. J. A. Biegler, as well suited to fill the Presidential chair. Elected unanimously.

Vice-President—Dr. H. C. Allen nominated Dr. W. J. H. Emory, of Toronto. Dr. Kent nominated Dr. Dillingham, and Dr. Butler, Dr. Custis.

President appointed Drs. Nash and Carr as tellers to count ballots. First ballot showed Dr. Emory 8, Dillingham, 6, and Dr. Custis, 12. Upon a second ballot Dr. Custis was elected unanimously Vice-President.

Secretary—Dr. S. A. Kimball was re-elected. Good!

Treasurer—Dr. C. W. Butler, Montclair, New Jersey.

Board of Censors—Drs. Schmitt, Bell, Gee, Rushmore, and Wesselhœft.

Dr. Dillingham moved for an investigation by the Board of Censors in regard to the charges brought against Dr. T. T. Oliver, of Chicago, for irregular practices. Referred to Board of Censors.

Dr. Stow offered this resolution:

"The I. H. A. tenders its congratulations to its colleagues of Rochester for their efforts to establish a Hahnemannian Hospital in Rochester, and views it with especial pleasure and gratification as one of the first institutions devoted to the practice of pure Homœopathy." Unanimously adopted.

Dr. Hitchcock moved that this resolution be furnished to the Rochester papers for publication. *Carried.*

Dr. Reed made a report in connection with the Homœopathic College of St. Louis, claiming that pure Homœopathy was being taught there, and the principles of the *Organon.*

Bureau of Obstetrics (Dr. Guernsey, Chairman) reported seven papers received from different members.

Dr. Butler read his paper, "Transverse Presentation," relating the extraordinary effects of Puls.$^{1m}$ in rectifying the mal-position.

Discussion by Drs. H. C. Allen, Kent, Nash, J. V. Allen, Schmitt, and Long, relating other somewhat similar cases.

Dr. Custis read " An Interesting Case."

Dr. J. V. Allen read " Repertory of Labor and After-pains." Read by title and referred.

Dr. Schmitt—paper, " The Value of Strictly Homœopathic Treatment."

Discussion on Dr. Schmitt's paper, by Drs. Long and Schmitt.

Motion of adjournment carried. Report of Bureau postponed.

Two P. M.—Dr. J. A. Biegler in the chair.

Dr. Guernsey read a paper on " Mastitis ;" recommended Lac caninum and Phytolacca in indurated and suppurative breasts, giving distinctions for their use.

Discussion by Drs. Allen (H. C.), Sawyer, Guernsey, Emory, Campbell. Dr. Baylies recommended also use of Graphites for old indurated cicatrices and breasts liable to frequent suppuration.

Paper by H. W. Brant, " Medicines in Parturition," read by title and referred to Committee on Publication.

Dr. Custis read paper entitled, " The Care of the Breasts," speaking principally of local application, to harden them previous to nursing.

Discussion by Drs. Biegler, Guernsey, Sawyer, Nash, H. C. Allen, Bell, Schmitt, Campbell, Emory. Dr. Reed disapproving of local applications, except the indicated remedy in high potency. Dr. H. C. Allen makes the local applications to the mother-in-law instead of patient !

Dr. Biegler recommended a memorial hour for report of Necrologist. *Carried.*

Bureau of Clinical Medicine (Dr. C. W. Butler, Chairman). Dr. Butler reported nine papers ; the first on the list was " Contributions to Materia Medica," by Dr. Fincke, of Brooklyn.

Motion by Dr. Emory that Dr. Fincke's paper be read and referred to Publishing Committee. *Carried.*

Paper by Dr. Kimball on " Syphilitis," where Belladonna was indicated remedy, but produced no effect when given dry, though a good effect followed when taken in water.

Discussion by Drs. Biegler, Stow, Nash, Dillingham, Sawyer, Reed, Campbell, E. T. Adams, and Schmitt.

Dr. Baylies read Dr. Fincke's paper.

Discussion by Drs. Emory, Reed, Butler, Schmitt, Adams, Custis, Dillingham. It was moved that a vote of thanks be tendered Dr. Fincke for his able paper, and a request be extended to him to become a member of this Society, and to present next year his further views and observations as to the results of the remedies and their potencies. *Carried.*

Paper by Dr. Butler, "Clinical Reports in their Relation to Homœopathy."

Discussed by Drs. Nash, Biegler, Emory, and Butler.

Evening Session, eight P. M. Dr. Biegler in chair.

Necrology.—Dr. Stow, Chairman, read the Report, which recorded the death of three members since the last meeting, held at Niagara Falls, viz.: Dr. Adolphus Felger, Dr. Theo. S. Keith, Dr. Geo. F. Foote.

Dr. Bell made some remarks in connection with the memory of Dr. Keith. The Report was very full, and showed the deceased members to have been of very high standing in their profession.

Bureau of Clinical Medicine then continued its report.

Paper by Dr. Sawyer relating to the action of Nux vomica in cases treated by "regulars," and Sulphur in cases of suppressed psora.

Discussion by Drs. Reed, Kimball, Evans, Butler (thought it a mistake to give Nux-vom.); Dillingham (gives it after mongrel treatment); Dr. Campbell (discarded that treatment, Puls. is often indicated); Sawyer.

Report of case of Dr. Oliver, of Chicago, referred to Censors.

Dr. Butler—He uses several remedies in rapid alternation; he is a spiritualist and employs two mediums.

Dr. Dillingham moved that Dr. Oliver be furnished with a copy of the charges, and, if not properly answered, to expel him, and that his name be referred to the Board of Censors, and if charges are not sustained to clear his reputation. *Carried.*

Place of next meeting not decided upon; the committee previously appointed was discharged and another, consisting of Drs. Biegler, Kimball, and Ballard, was appointed, and they were ordered to report their choice within thirty-five days.

Bureau of Clinical Medicine—Dr. Dillingham was appointed chairman of this bureau.

Dr. Butler moved for a vote of thanks by the Association to Dr. Tyrrell, late Treasurer, for the excellent work done during the past year. *Carried.*

*Reports of Delegates.*—Dr. Sawyer, delegate from the Indiana Institute of Homœopathy, reported their endeavors to obtain one of the State insane asylums but failed; another to be made.

There are about three hundred so-called homœopathic physicians, and, possibly, half a dozen real "homœopathists," but there is a revival going on there.

Dr. Ballard referred to the report of Dr. Foote having gone into physical and mental science; he looked into it but did not practice it.

*Resolutions.*—Dr. Schmitt—Mr. President, I move that we pass a vote of thanks to the members from Toronto for receiving us so well, also to the host of this hotel for his kind accommodation. *Carried.*

Dr. Stow—I move that the thanks of this Association be extended to the officers who have so faithfully served us during the past year. *Carried.*

Motion for adjournment (*sine die*). *Carried.*

Ten P. M., June 20th, 1889.

S. A. KIMBALL, *Secretary.*
Per R. HEARN, *Assistant Secretary.*

